# *Mycobacterium bovis* shedding patterns from experimentally infected calves and the effect of concurrent infection with bovine viral diarrhoea virus

**DOI:** 10.1098/rsif.2006.0190

**Published:** 2006-12-19

**Authors:** R.R Kao, M.B Gravenor, B Charleston, J.C Hope, M Martin, C.J Howard

**Affiliations:** 1 Department of Zoology, University of OxfordSouth Parks Road, Oxford OX1 3PS, UK; 2 University of Wales at Swansea, School of MedicineSwansea SA2 8PP, UK; 3 Institute for Animal Health, Compton LaboratoryCompton RG20 7NN, UK

**Keywords:** mathematical model, bacteria, macrophages, tuberculosis

## Abstract

Concurrent infection of cattle with bovine viral diarrhoea virus (BVDV) and *Mycobacterium bovis* is considered to be a possible risk factor for onward transmission of bovine tuberculosis (BTB) in infected cattle and is known to compromise diagnostic tests. A comparison is made here of *M. bovis* shedding (i.e. release) characteristics from 12 calves, six experimentally co-infected with BVDV and six infected with *M. bovis* alone, using simple models of bacterial replication. These statistical and mathematical models account for the intermittent or episodic nature of shedding, the dynamics of within-host bacterial proliferation and the sampling distribution from a given shedding episode. We show that while there are distinct differences among the shedding patterns of calves given the same infecting dose, there is no statistically significant difference between the two groups of calves. Such differences as there are, can be explained solely in terms of the shedding frequency, but with all calves potentially excreting the same amount of bacteria in a given shedding episode post-infection. The model can be thought of as a process of the bacteria becoming established in a number of discrete foci of colonization, rather than as a more generalized infection of the respiratory tract. In this case, the variability in the shedding patterns of the infected calves can be explained solely by differences in the number of foci established and shedding being from individual foci over time. Should maximum exposure on a particular occasion be a critical consideration for cattle-to-cattle transmission of BTB, cattle that shed only intermittently may still make an important contribution to the spread and persistence of the disease.

## 1. Introduction

Infection of domesticated cattle with *Mycobacterium bovis* or bovine tuberculosis (BTB) is a global problem, important both owing to its economic cost and because it is a zoonosis. While the introduction of standardized testing regimes has led to the eradication or dramatic decline of BTB incidence in many countries, BTB remains a problem in a number of them. Notably, despite a comprehensive testing regime and enormous resources spent on its control, BTB in the UK has not only persisted but has also been on the increase for at least the past 30 years (Krebs *et al*. 1997 and http://www.defra.gov.uk/animalh/tb/stats/index.htm). A general concept is that the eradication of disease is not possible for those infectious agents that persist in a wild animal reservoir. The strong correlation between areas with high prevalence of BTB in the badger (*Meles meles*) and a high incidence of BTB in the cattle supports the argument that the two are related and that this reservoir of infection is a major reason for the continuing infection with *M. bovis* in the UK herd (Griffin & Dolan 1995; Krebs *et al*. 1997; Gallagher & Clifton-Hadley 2000; Woodroffe 2006). In New Zealand, the possum has been identified as the major source of infection for cattle and eradication has not proven possible (Coleman & Cooke 2001). However, control of BTB in badgers is problematic (Donnelly *et al*. 2003), and the relative roles of cattle-to-cattle and badger-to-cattle Tb transmission in the persistence and spread of BTB from herd-to-herd are uncertain. There is experimental evidence supporting the view that cattle-to-cattle spread does occur (Neill *et al*. 1989; Costello *et al*. 1998; Menzies & Neill 2000) and this may be important in the maintenance and spread of BTB (Gilbert *et al*. 2005; Gopal *et al*. 2006). Bovine viral diarrhoea virus (BVDV) is believed to be widespread in the UK national herd (Paton *et al*. 1998). There is anecdotal evidence that concurrent infection with BVDV and *M. bovis* can increase the severity of BTB (Monies 1999), and experimental data suggest that it may compromise diagnostic tests for BTB (Charleston *et al*. 2001). Therefore, we examine quantitative data describing the amount of bacterial shedding (i.e. release) from calves following experimental infection, where half of the calves that had been infected with *M. bovis* were concurrently infected with BVDV, and the other half not. Some characteristics of *M. bovis* shedding have previously been described, including randomness and intermittency, with bacteria shed in individual bursts or episodes (Francis 1947; Neill *et al*. 1988, 1989); however, no previous report has described or analysed data that include measures of the quantity of bacteria shed.

While no relationship between amount of shedding and infectivity has been established, it seems reasonable that a positive relationship will exist between bacterial shedding and the potential for an infected animal to infect others. Previous reports have modelled the cattle-to-cattle transmission process by assuming that all infected cattle will test positive and contribute equally to the transmission process (Barlow *et al*. 1996; Kao *et al*.1997; Kao & Roberts 1999). Should there be significant differences in either or both of these factors, then it may be that some of the infected cattle make a much greater contribution to transmission than others.

## 2. Methods

### 2.1 Bacteria and virus

*Mycobacterium bovis* strain, AF 2122/97, was grown in Middlebrook 7H9 broth (containing 10% ADC supplement) for 7 days and aliquots of log-phase organisms frozen at −70°C. The number of colony-forming units (CFUs) was determined by titration on modified 7H11 agar (containing 10% OADC supplement and 4.16 g pyruvate per litre) and incubated aerobically at 37°C for four weeks. This strain is a fully virulent Great Britain strain isolated in 1997 from a diseased cow suffering from caseous lesions in the lung and bronchomediastinal lymph nodes, the genome sequence for which is available (Vordermeier *et al*. 2002). BVDV strain, ncpBVDV 11249, was used for the concurrent infection. This strain is a field isolate causing clinical signs, viraemia and nasal shedding consistent with field infections. Challenge protocols, except the route of challenge (intranasal in the cited publication) and analysis of viral infection, were performed as described in Charleston *et al*. (2001). A 1 ml aliquot was thawed and diluted to give the appropriate dose for inoculation. The number of CFUs in the inoculum was confirmed by titration on 7H11 agar.

### 2.2 Calves and infection model

Subjects were conventionally reared Friesian cross calves that were from a farm that had been free of BTB for more than 10 years and were free of *M. bovis* infection as judged by responses when tested in the whole-blood IFNγ test (Wood & Jones 2001). These calves were housed in a high security unit in four groups of three. All 12 calves were infected with 10^6^ CFU of *M. bovis* on day 0. The calves were infected with two 1 ml volumes of bacterial suspension by the intranasal route via a cannula of about 5 cm with 1 ml being delivered into each nostril (Neill *et al*. 1988). On day 7, six of the calves (labelled 1–6 in figure 1) were infected subcutaneously with 5×10^6^ TCID50 non-cytopathogenic BVDV strain 11249 (Charleston *et al*. 2001). Nasal secretions, about 0.5 ml, were collected from the anterior nares of calves via a plastic cannula by means of non-invasive sterile samplers (Bibby Sterilin, Staffordshire, UK) and application of a negative pressure with a hand pump. Mucus was made up to 2 ml with sterile phosphate-buffered saline.

Samples of nasal secretions that had been taken twice a week for eight weeks were frozen at −70°C for subsequent bacteriological examination and were cultured on modified 7H11 agar without decontamination. All cultures were incubated aerobically at 37°C for four weeks and numbers of colonies counted. Suspect isolates were examined by microscopy after Ziehl–Neelsen staining. Acid-fast isolates showing typical colonial morphology were assumed to be *M. bovis*.

The experiment was approved by the local ethics committee according to the national UK guidelines.

## 3. Analysis

### 3.1 Statistical methods

A set of linear mixed models were constructed using calves as experimental units. Components of the model were analysed to determine whether there were significant differences in the shedding profiles between the two groups. Data were log-transformed before analysis and all models were constructed using the statistics package R (Ihaka & Gentleman 1996).

### 3.2 Models

Interpretation of these data is confounded by the problem of sampling frequency. As samples are taken only every few days, it is possible that the differences between calves may simply be due to random variation in the sampling timeframe. Therefore, we explore the hypothesis that the shedding of bacteria differs only in frequency of excretion using a simple model. This model consists of three independent components: the frequency of shedding burst; the profile of individual shedding bursts over time; and the magnitude of shedding bursts.

### 3.2.1 Modelling shedding frequency

First, we assume that shedding of bacteria is associated with the bursting of individual lesions. This is modelled as a random event described by a Poisson distribution, defined by

(3.1a) $$p(n)=\frac{{\text{e}}^{-\lambda (t)}\lambda {\left(t\right)}^{n}}{n!},$$

(3.1b) $$P(x)=\sum_{x=0}^{n}p(n),$$

where *x* and *n* are non-negative integers, *λ*(*t*) is the frequency of shedding at time *t*, *p*(*n*) is the probability that n events will occur and thus *P*(*x*) is the cumulative probability that there are *x* or fewer shedding events producing measurable quantities of bacteria when a sample is taken at time *t*. Thus, if *x*=1, for example, equation (3.1*b*) describes the probability that 0 or 1 event will occur. Two different models are considered. In the first, *λ*(*t*) is assumed constant (one-phase model), and in the second *λ*(*t*) is assumed to start at zero and then is a constant positive value thereafter (two-phase model), i.e.

(3.2) $$\lambda (t)=\{\begin{array}{cc} 0 & t\leq t_{0}\\ \lambda_{0} & t>t_{0} \end{array},$$

where *λ*_0_ is the constant frequency after time *t*_0_.

### 3.2.2 Modelling shedding burst magnitude

The second component of the model is a fit of the average shedding profile using a simple mathematical model of bacterial replication. Here, we assume that bacteria invade and multiply at an exponential rate, and thus when shedding occurs, are released at an average rate *B*(*t*). Bacterial infection and replication are initially controlled by the cell-mediated immune response, with bacteria phagocytosed by macrophages (*M*(*t*)), which proliferate at a rate that depends on the density of bacteria (Perez *et al*. 2002). If removal of macrophages over the short time frame of the experiment is ignored, then average bacterial shedding can be described by a simple two-variable model,

(3.3a) $$\frac{\text{d}B}{\text{d}t}=\gamma B-\rho BM,$$

(3.3b) $$\frac{\text{d}M}{\text{d}t}=\lambda B.$$

Further discussion of the models as they relate to the data is found in the results.

### 3.2.3 Modelling individual shedding profiles

If excretion is the sum of individual episodes of shedding, and each episode experiences a distinct rise and fall with finite slope, then the time past the start of the shedding episode at which a sample is taken is important for determining the quantity of bacteria recovered. Therefore, it is unlikely that the amount of bacteria recovered directly represents either the maximum shedding or the total amount released. Accordingly, we reconstruct a typical episode profile by fitting the distribution of sampling at a given time with a lognormal distribution, described by

(3.4) $$\Phi \left(\frac{\text{log}(S(t))}{\text{log}(B(t))}\right)=\frac{1}{\sqrt{2\pi }}\int_{-\infty }^{\text{log}(S(t))/\text{log}(B(t))}{\text{e}}^{-{\left(y-1\right)}^{2}}\text{d}y,$$

where *S*(*t*) is the amount of bacteria recorded in a sample at time *t*; *ϕ* (log(*S*(*t*))/log(*B*(*t*))) (logs are base 10) is the cumulative probability that a sample containing *S*(*t*) CFU ml^−1^ will be found when the average shedding level is *B*(*t*) CFU ml^−1^. Technically, this allows for bacterial shedding of less than zero, but in practice the variance of the distribution is narrow enough for this to be unimportant. Data at some time points are too sparse (i.e. none) to generate typical profiles or to make any meaningful comparisons between time points. Therefore, we normalize the data at each time *t* using the expected mean shedding quantity at time *t* from the solution of the system of equations (3.3*a*) and (3.3*b*). We assume that the shape of the profile does not change over the course of the experiment, and so for samples taken at any given time *t*, the ratio log(*B* (*t*))/*σ*(*t*) is constant, where *σ*(*t*) is the standard deviation of log(*S*(*t*)) at time *t*. In other words, if the maximum shedding level in one event is 100 CFUs per ml, for example, and the maximum in another is 1000, then the probability of collecting a sample when the current shedding level is 10 CFUs per ml in the first case is the same as the probability of collecting when the shedding level is 100 CFUs per ml in the second.

## 4. Results

### 4.1 Identifying differences in shedding patterns between the two infection groups

The simplest analysis of the repeated measures data was to include a fixed effect for BVDV and a single random effect (intercept) for each calf (figure 1). The estimated effect of BVDV is to increase the daily shedding by an average of 2.2 CFU, a non-significant increase (*p*=0.42) which is reflected in the wide 95% CIs (−0.63, 12.2). The random effects due to calf are not of primary interest here, but do show a similar within-calf variance in comparison to between-calf variance.

To explicitly model the time element of shedding profiles, we included a ‘day’ covariate. A good model for the data was found to be a quadratic shedding profile with random effects terms for both the linear and the quadratic terms for each calf. This accounts for between-calf variation in both the timing of peak shedding and the curvature at the peak. The model provided a significantly better fit than all nested models with simpler fixed or random effects (see fit in figure 1). With respect to the fixed effect of BVDV, however, the conclusions remained the same as the basic analysis; there was no significant increase in mean CFU shedding in the BVDV-infected group (*p*=0.72).

### 4.2 Analysing the shedding characteristics

Whether or not there are differences between the two groups, there remains the question of what the source of those differences might be. A more detailed examination of the temporal dynamics in the raw data (figure 1) reveals some patterns. First, shedding of bacteria is intermittent, with bacteria recovered on some days and not on others. The intermittency appears to be calf dependent, with some calves shedding on nearly every sampled day and some shedding relatively rarely. Second, there appears to be a rise and fall in the amount shed per sample taken over the course of the experiment.

As no significant difference was found between the two groups, the data from all 12 calves were combined in the analyses to give the maximum amount of raw data. These data were examined to determine if, in addition to the evidence for differences in the frequency of shedding, there is also evidence for differences in the maximum amount of shedding.

As the models are nested, the likelihood ratio test is used to determine the appropriate model (one-phase or two-phase) for each shedding pattern. In 4 of the 12 calves (IDs 1, 2, 4 and 9), the two-phase model was significantly better at the *p*=0.05 level, with a fifth (ID 6) at the *p*=0.10 level (table 1). The normalised shedding profile from these defined by equation (3.4) is shown in figure 2. This group corresponds to calves with higher frequencies of shedding. Fitting the frequency of shedding using equations (3.1*a*) and (3.1*b*), calves can be classified as intermittent shedders (one-phase calves) or persistent shedders (two-phase calves). For calf 5, no shedding was detected, thus the expected frequency of shedding is zero, but there is an upper bound and only a one-phase model is consistent. While calves 3, 7 and 8 are qualitatively of similar type to the persistent shedders, models based on equation (3.2) cannot be used to determine the expected value of *λ*: since shed bacteria are detected for every sample past *t*_0_ (even though resampling might detect a gap), *λ*=∞ will give the best fit (note that for calf 3, no sample was taken on day 24).

The equations (3.3*a*) and (3.3*b*) describing the mean shedding profile were then fit to the average amount shed, considering only calves with non-zero shedding at each time point. The maximum-likelihood fit of the model appears to provide a reasonable fit to the average of the data (figure 3). Since the data are shown on a logarithmic scale, at early time points the log of the averages is less than zero (with a limit of negative infinity when there is no shedding), so here are set to zero for convenience. Note that there is considerable variation (over three orders of magnitude) in the amount recovered from different calves.

In order to compare sampling at each time point, a typical shedding profile for lesions bursting at each time *t* is required. The distribution is in general a combination of differences between the calves and differences in the sampling point in the shedding episode. The hypothesis explored here is equivalent to stating that differences between calves are mainly due to the differences in the frequency of episodes and that apparent differences in the episodes themselves are due to the time at which samples were taken. The fitted shedding profile is shown in figure 2. The extreme shedding counts on the right are both cases where there were only one datum at the given time point (days 7 and 55), and thus are likely to be an artefact of the normalization procedure.

The frequency with 95% CIs is shown in table 1. Simulated shedding profiles at time *t* are then generated by a stochastic model described by

(4.1) $$S(t)=B(t)\times \text{log}\left(\sum_{i=1}^{P^{-1}(R_{1})}{\text{e}}^{\Phi^{-1}}(R_{\text{i}})\right),$$

where *S*(*t*) is the amount of bacteria sampled, *R*_1_ and *R*_2_ are random numbers from 0 to 1, *B*(*t*) is the solution to equations (3.3*a*) and (3.3*b*), and *ϕ*^−1^(*R*_1_) and *p*^−1^(*R*_1_) are the inversion functions of equations (3.4), (3.1*a*) and (3.1*b*), respectively.

Using the shedding profile parameters for equations (3.3*a*) and (3.3*b*) for those calves classified as ‘persistent shedders’ and the best-fit frequency of shedding for the ‘intermittent shedders’, 200 simulations are run, generating measured profiles for each day samples were taken in the actual experiment. Ranking simulations in terms of the total amount of shed bacteria over all episodes per simulation, excluding the top five and the bottom five simulations, then represent estimates of the 95% CIs for bacterial shedding. In figure 4, the experimental data for all calves classified as intermittent shedders are compared with the outputs using the frequency model that best corresponds to those data, in combination with the episode model generated from the dataset comprising all persistent shedding calves. In all cases, the intermittent shedders fit well within the estimated 95% CIs for the model over the entire time course measured.

## 5. Discussion

Considerable variation was evident for calves with respect to quantity of *M. bovis* recovered from nasal secretions. Consideration needs to be given for whether this was due to a genuine biological phenomenon or whether it was simply a technical consequence of some unknown variability in the performance of the sampling and assays. There was a highly significant linear correlation between the extent of the lesions (lesion score as defined in Stone 1979) evident when calves were examined post-mortem and the total number of bacteria collected (*R*^2^=0.636, *p*=0.002) and between the extent of lesions and the number of days on which bacteria were shed (*R*^2^=0.522, *p*=0.008). Thus, it is likely that the shedding of bacteria in nasal secretions reflects the level of disease in individual calves due to variation in resistance, which is either genetic, i.e. innate or adaptive and the result of experience of other infections.

It also appears that the overall level of shedding increases for the first four weeks after exposure and then begins to decline. This is consistent with the appearance of the immune response after infection and high levels of IFNγ (Buddle *et al*. 1995; Charleston *et al*. 2001). However, shedding clearly continues after the time when IFNγ is known to be produced by infected calves. Thus, from the reduced bacterial shedding, it appears that the immune response partially controls the infection but does not clear it.

Should co-infection with BVDV result in greater shedding or a higher probability of transmission, this would be an important risk factor to be considered in an epidemiological control programme. Unfortunately, though there was some evidence for a difference between the two groups, it was not statistically significant. The power for detecting a significant BVDV effect of the size estimated from the mixed models is clearly low, given the high coefficient of variation in CFU shedding. An experiment able to detect a moderate increase in shedding with high power would require large group sizes (30–50). Taking into account the practical constraints on group size which are often faced in this field, it is important that the meta-analysis across similar experiments and the pooling of data from different investigations are first undertaken. Quantitative descriptions, such as are provided here, will aid such analyses, as well as further experimental design.

The data are consistent with a model in which exposure results in the establishment of individual foci of infection and calves with more extensive disease have more foci. These individual foci then release bacteria into the respiratory tract and they are shed in the nasal secretions. These foci should not be regarded as being granulomas present later in the progression of BTB, which undergo the process of caseation and liquefaction that releases infectious material into the airways (Costello *et al*. 1998; Charleston *et al*. 2001). The analyses here show that differences in the shedding profiles were consistent with assuming that the only significant difference between intermittent and persistent shedders was the frequency with which shedding episodes occurred, i.e. that individual bursts of shedding of bacteria from foci produced very similar numbers of bacteria in nasal secretions on any particular occasion. Should the maximum numbers of bacteria shed on any single occasion be the principle indicator of infectiousness, and infection be more dependent on a susceptible calf receiving a single large dose, then infrequent shedders will still contribute to transmission of disease. Should, however, long-term and repeated exposure be important, the contribution of intermittent shedders may be less significant. Variability in response could have implications for the design of future experiments, models of targeted disease control or the development of a cattle vaccine. It may determine, for example, whether it is more important to reduce transmission from those cattle most probably to shed bacteria, or reducing the shedding rate of all infected cattle, or whether cattle partially protected by vaccine remain a risk for transmitting disease. Depending on which model of infectious contact is more correct, this could have wide-ranging implications for the control of disease and needs to be explored with appropriate dose–response experiments. All cattle testing positive for *M. bovis* are currently removed from the herd as soon as possible, and there is no recommendation that this should change. Differences in shedding could be due to chance, or phenotypic or genotypic differences in the calves. Prior exposure to environmental organisms has been reported to impart a degree of resistance to tuberculosis in humans and in animal models designed to mimic this (Fine 1995). Furthermore, genetic differences in the NRAMP1 gene have been related to resistance to tuberculosis in humans and rodent models (Krebs *et al*. 1997; Bellamy *et al*. 1998). Should some cattle be phenotypically or genetically predisposed to shedding large numbers of bacteria into the environment over long periods of time, then to improve the control procedures it would be important to identify them and remove them from the herd quickly in the short term and, in the latter case, from the breeding pool in the longer term.

## Figures and Tables

**Figure 1:**
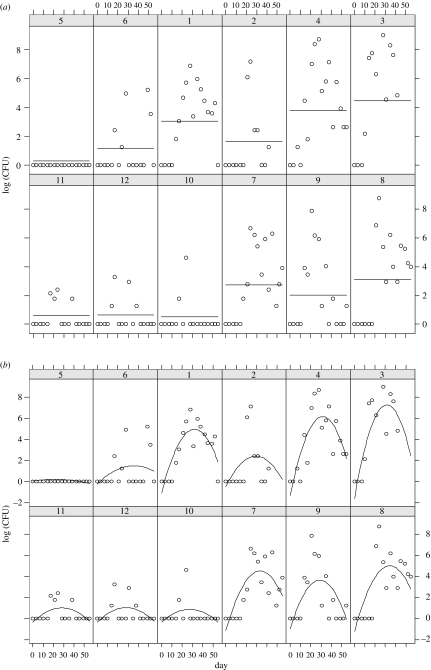
Raw data from 12 calves, number 1–12. Horizontal axis in days, vertical access in log(CFU ml^−1^). Calves 1–6 were concurrently infected with *M. bovis* and BVDV, calves 7–12 infected with *M. bovis* only. The two figures represent fitting of the two statistical models to the data. (*a*) Fit of mixed model with fixed effect of BVDV and random intercept for each calf. (*b*) Fit of mixed model with fixed effect of BVDV and random effects: intercept, slope and curvature for each calf.

**Figure 2:**
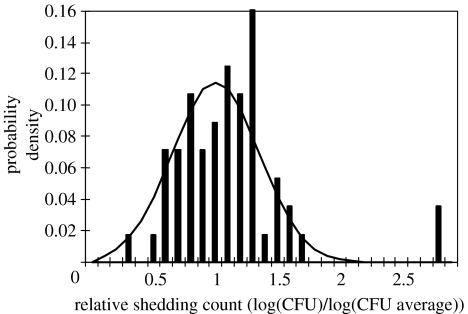
Fit of a normal distribution to the sample data. Data are taken from all calves fitting the two-phase (persistent) shedding model (calves 1, 2, 4, 6 and 9). Data from any single time point is normalized by dividing by the average sample count, and the data from all time points then pooled to create the profile.

**Figure 3:**
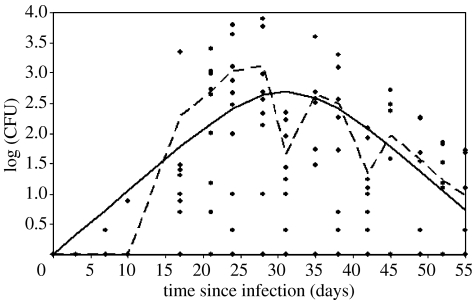
Fit of model of bacterial growth (solid line, equation (3.4)) to average measured log (CFU) (dashed line). Data points from all calves in the experiment shown to illustrate the spread in the data.

**Figure 4:**
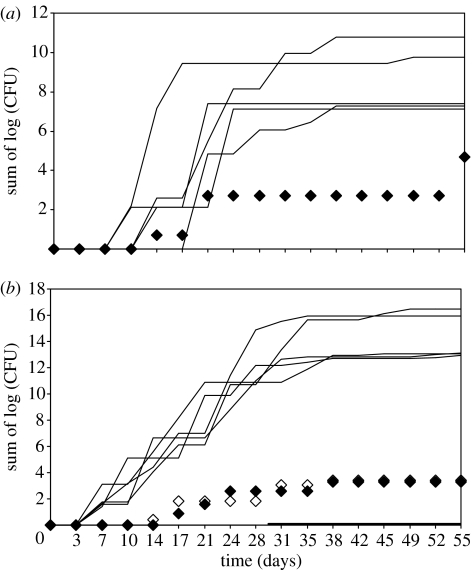
Simulations showing fits of (*a*) calf 10 and (*b*) calves 11 (hollow diamonds) and 12 (solid diamonds). Two hundred simulations were run. All shedding patterns were well within the 95% confidence bounds of the model, indicated by the individual simulations with the five highest totals and five lowest totals (black lines). In figure 4*a*, none of the five lowest totals have a cumulative shedding that is non-zero.

**Table 1:** Fitted frequency values. Data are fit to two models, the first where shedding occurs at fixed frequency throughout the observed period (one-phase or 1P) and the second where there is a shedding time delay until the first measured occurrence, after which shedding occurs at fixed frequency (two-phase or 2P). The likelihood ratio test is used to identify when the 2P model is significantly better (in bold, *p*<0.10). Frequencies shown are respectively the fixed frequency and the fixed value after the time delay (95% CIs in brackets). CBD indicates ‘cannot be determined’, i.e. calves for which the two-phase model is clearly more appropriate, but no mean frequency could be determined.

ID number	model	frequency	LTR Test
1	two-phase	2.56 (0.56, 10.7)	**5.9**
2	two-phase	1.25 (0.79, 1.88)	**3.9**
3	CBD		
4	two-phase	2.30 (1.58, 3.33)	**3.9**
5	one-phase	0.0 (0.0, 0.24)	
6	two-phase	0.45 (0.28, 0.69)	**2.7**
7	CBD		
8	CBD		
9	two-phase	1.1 (0.77, 1.5)	**3.6**
10	one-phase	0.12 (0.06, 0.22)	0.7
11	one-phase	0.27 (0.16, 0.41)	1.4
12	one-phase	0.27 (0.16, 0.41)	1.1
